# Efficacy and safety of anlotinib combined with ^125^I seed implantation for iodine-refractory thyroid cancer

**DOI:** 10.3389/fendo.2025.1587412

**Published:** 2025-08-22

**Authors:** Zhijun Chen, Xinlan Tang, Liling Tan, Yu Su, Wenjun Wang, Zhen Wu

**Affiliations:** ^1^ Department of Nuclear Medicine, Jiangxi Cancer Hospital, Nanchang, Jiangxi, China; ^2^ Nanchang University, Nanchang, Jiangxi, China; ^3^ Department of Nuclear Medicine, The Second Affiliated Hospital of Nanchang University, Nanchang, Jiangxi, China

**Keywords:** radioactive iodine-refractory thyroid cancer, anlotinib, iodine-125 seed brachytherapy, combination therapy, multikinase inhibitor, progression-free survival, targeted therapy

## Abstract

**Background and objective:**

Radioiodine-refractory differentiated thyroid cancer (RAIR-DTC) remains challenging to treat due to a lack of effective therapies. This study aimed to evaluate the efficacy and safety of combining anlotinib with iodine-125 (^125^I) seed implantation in patients with RAIR-DTC.

**Methods and materials:**

We retrospectively compared three treatment groups in 52 patients with advanced RAIR-DTC: anlotinib monotherapy (Group A, *n* = 14), ^125^I seed brachytherapy monotherapy (Group B, *n* = 25), and combined therapy (Group C, *n* = 13). Clinical outcomes including local progression-free survival (LPFS), overall survival (OS), tumor response, serum thyroglobulin (Tg) levels, and adverse events were analyzed.

**Results:**

As of February 2025, the combination therapy group achieved a longer median LPFS (42.2 months) than either monotherapy group (18.6–18.7 months; *p* = 0.023) and a higher objective response rate at 6 months (77% vs. 21–32% with monotherapies; *p* < 0.05). Tumor volumes in all groups decreased after treatment, with the greatest reduction within 6 months in the combination group (*p* < 0.001). By 12 months, response differences between groups narrowed, and median OS was similar across groups (~22–43 months, *p* = 0.425). Serum Tg levels declined significantly from baseline in all groups. No major procedural complications occurred, and treatment-related adverse reactions were mostly mild (Grade 1–2) and comparable among groups.

**Conclusion:**

Combining ^125^I seed brachytherapy with anlotinib demonstrated superior short-term tumor control and prolonged local disease remission in RAIR-DTC, without increasing toxicity. This combination may offer a promising therapeutic option for RAIR-DTC, pending further validation in larger studies.

**Clinical trial registration:**

https://clinicaltrials.gov, identifier NCT06362772.

## 1 Introduction

Thyroid cancer is the most common malignant tumor of the endocrine system, with incidence and mortality increasing steadily over the past four decades (1). As of 2022, thyroid cancer became the third most common malignancy in China, after lung and colorectal cancers (2). Differentiated thyroid carcinomas (DTCs), including papillary and follicular thyroid carcinomas (PTC and FTC), account for approximately 90% of thyroid cancers (3). Most DTCs can achieve remission through surgery, radioiodine (¹³¹I) therapy, and thyroid-stimulating hormone suppression therapy. However, metastatic disease occurs in 5–10% of DTC cases, and around two-thirds of these metastases eventually become radioiodine-refractory (4). Patients with radioiodine-refractory DTC (RAIR-DTC) often exhibit rapid tumor progression and poor prognosis, with a 10-year survival rate of less than 10%. Currently, effective therapeutic options for this patient population are very limited.

Angiogenesis plays a critical role in the progression and metastasis of thyroid cancer. Overexpression of vascular endothelial growth factor (VEGF) and its receptors has been observed in thyroid cancer cells, promoting tumor growth and neovascularization (5). Multikinase inhibitors (MKIs) such as sorafenib and lenvatinib have been approved for treating RAIR-DTC, demonstrating improved progression-free survival (PFS). However, challenges including drug resistance, adverse side effects, and limited patient eligibility persist. Anlotinib, a novel oral MKI, inhibits multiple targets including VEGFR, PDGFR, FGFR, c-Kit, and RET (6), and it offers the advantage of milder adverse reactions compared to other tyrosine kinase inhibitors (TKIs). Anlotinib has shown favorable efficacy and safety in treating recurrent or metastatic RAIR-DTC and has been approved in China for progressive RAIR-DTC (7). Nevertheless, in clinical practice we have found that anlotinib has limited efficacy in controlling large metastatic tumors, which has emerged as a new challenge in the management of RAIR-DTC.

^125^I seed implantation therapy, also known as ^125^I brachytherapy, involves implanting radioactive seeds that continuously release low-dose γ-rays, inducing DNA damage (e.g., hypomethylation) and radiation-induced tumor cell apoptosis (8). The emitted γ-rays have low penetrability— a 1 mm lead plate can block over 99% of the radiation (9)—making this therapy relatively safe for normal tissues adjacent to the tumor as well as for healthcare providers during the implantation procedure. Compared with conventional external-beam radiotherapy, interstitial implantation of ^125^I seeds directly into tumor tissue or metastatic sites delivers a high radiation dose to the target area while minimizing exposure to surrounding normal tissues. This approach provides a highly efficient radiation dose distribution, favorable radiobiological properties, reliable clinical efficacy, and minimal damage to adjacent organs. Multiple studies have reported excellent local tumor control achieved by ^125^I seed implantation in RAIR-DTC patients (10–13).

Combining anlotinib with radiotherapy has demonstrated high efficacy, feasibility, and safety in other settings. For example, Shi et al. (14) reported that in a patient-derived xenograft mouse model of esophageal cancer, the group treated with anlotinib plus radiotherapy showed significantly greater tumor growth inhibition than groups receiving control, radiotherapy alone, or radiotherapy plus cisplatin. Moreover, the combination therapy did not induce severe adverse reactions in the mice. Tang et al. (15) described a case of postoperative recurrent retroperitoneal liposarcoma treated with ^125^I seed implantation combined with anlotinib; a 3-year follow-up indicated stable disease control. Based on these findings, we hypothesized that in RAIR-DTC patients, combining ^125^I seed brachytherapy with anlotinib targeted therapy could achieve improved control of large local tumors while simultaneously treating systemic disease.

**Figure 1:**
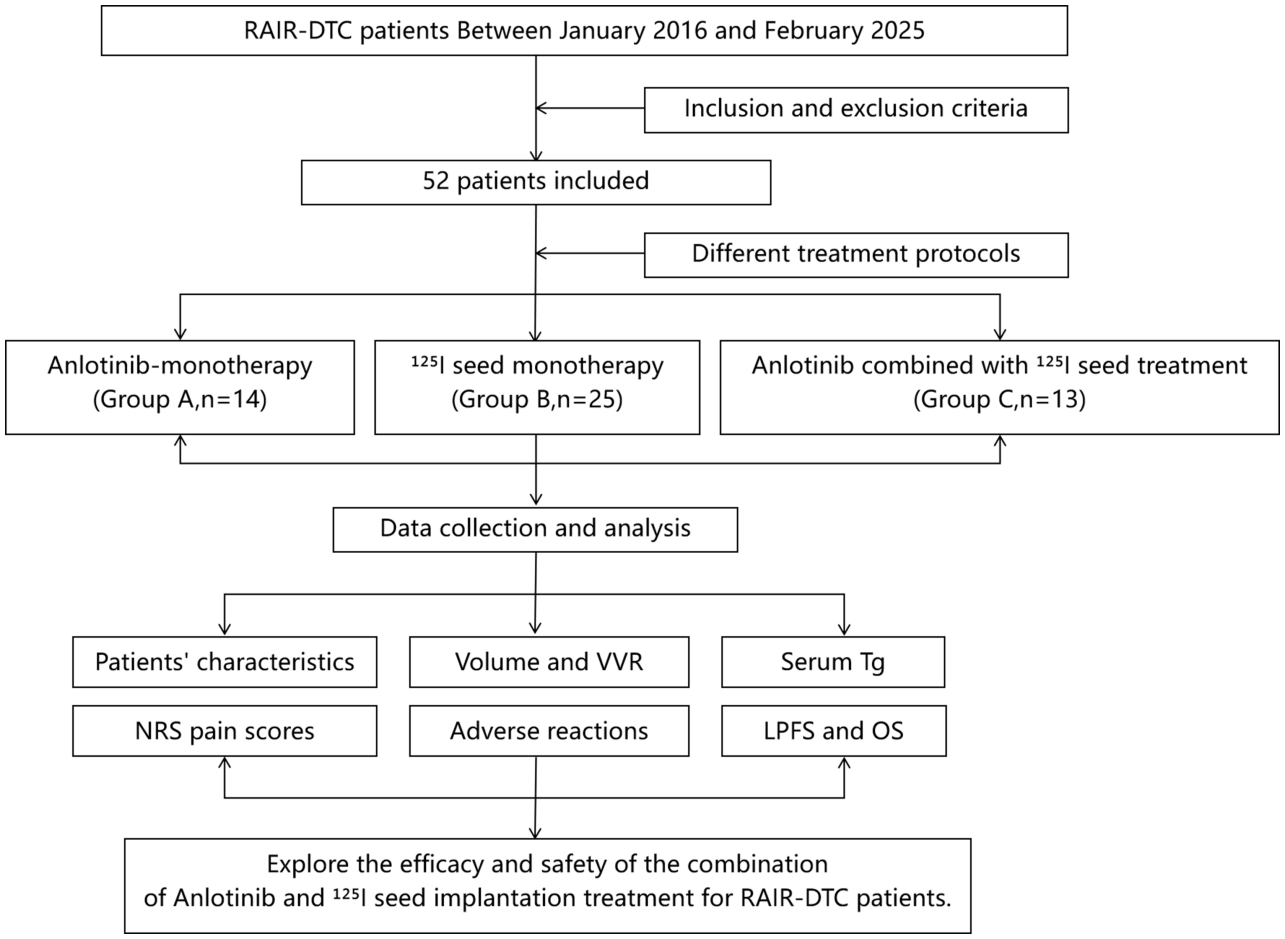
Flowchart of the study.

To our knowledge, this study is the first report on the use of ^125^I seed implantation combined with anlotinib for RAIR-DTC. We retrospectively collected clinical data for RAIR-DTC patients treated at Jiangxi Cancer Hospital from January 2016 to February 2025. According to the treatment regimens received, patients were divided into anlotinib-only treatment group (Group A), ^125^I seed implantation-only treatment group (Group B), and ^125^I seed brachytherapy combined with anlotinib group (Group C). We analyzed and compared the efficacy and safety outcomes of the three treatment approaches (**Figure 1**).

## 2 Materials and methods

### 2.1 Patient selection

Patients with RAIR-DTC who were treated at Jiangxi Cancer Hospital between January 2016 and February 2025 were retrospectively identified.

Inclusion criteria:

1. Meeting the diagnostic criteria for RAIR-DTC (according to Chinese guidelines (16)): (①) all known lesions show no significant radioiodine uptake; or (②) some lesions do uptake iodine, but disease progression occurs after ¹³¹I treatment.
2. Presence of measurable lesions, with at least one tumor lesion having a maximum diameter > 0.5 cm.
3. Expected survival time of more than 3 months.
4. Karnofsky Performance Status (KPS) score ≥ 80.

Exclusion criteria:

1. Positive thyroglobulin antibody (TgAb) or thyroid-stimulating hormone (TSH) ≥ 0.5 mU/L at baseline.
2. Incomplete clinical data.
3. Poor compliance with treatment or inability to cooperate with the seed implantation procedure.
4. Previous treatment with other MKIs such as lenvatinib or sorafenib.
5. Severe cardiopulmonary dysfunction (e.g., FEV_1_ < 30% of predicted; or left ventricular ejection fraction < 35%).
6. Severe coagulation disorders (platelet count < 20×10^9^/L or prothrombin time ratio > 1.5).
7. Inability to tolerate the side effects of targeted therapy.
8. Expected survival time < 3 months.

A total of 52 patients met the criteria and were included. They were categorized into the anlotinib-only group (Group A, *n* = 14), the ^125^I seed implantation-only group (Group B, *n* = 25), and the combined ^125^I seed implantation plus anlotinib group (Group C, *n* = 13), according to their treatment modality (see **Figure 1**). The study was approved by the Ethics Committee of Jiangxi Cancer Hospital. All patients were fully informed about their condition and treatment options—including the expected efficacy and possible adverse reactions of ^125^I seed implantation, anlotinib targeted therapy, the combination of both, as well as other treatments such as external radiotherapy or chemotherapy—and each provided written informed consent prior to treatment.

### 2.2 Materials and equipment

The treatment planning system (TPS) for seed implantation was provided by Beijing Tianhang Kelinzhong Technology Co., Ltd. The ^125^I seeds were provided by Beijing Atom High-Tech Biotechnology Co., Ltd., with an activity of 2.22–2.96 × 10^7^ Bq per seed. The implantation needles were 18G puncture needles produced by HAKKO Co., Japan. A fully automated Siemens (Germany) biochemical immunoassay analyzer was used to measure thyroid function indicators. The following laboratory indices were measured at baseline and at 2, 4, and 6 months post-treatment: free triiodothyronine (FT_3_), free thyroxine (FT_4_), TSH, thyroglobulin (Tg), and thyroglobulin antibody (TgAb).

### 2.3 Treatment methods

All patients received treatment and follow-up in the Department of Nuclear Medicine at our hospital.

^125^I Seed Implantation: Before the procedure, patients underwent evaluations including coagulation profile, liver and kidney function tests, cardiopulmonary function assessment, and imaging (CT) to plan the seed implantation. Physicians and physicists jointly developed an individualized treatment plan. The prescription dose for brachytherapy was 80–120 Gy. Specifically, for patients who had received external-beam radiotherapy within the past 6 months, a lower prescription dose of 80 Gy was chosen; for those who had received external radiotherapy more than 6 months prior or had a cumulative ¹³¹I treatment dose exceeding 2.22 × 10¹^0^ Bq, a dose of 100 Gy was used; and for all other patients, 120 Gy was prescribed. ^125^I seeds with appropriate radioactivity were selected according to the location of metastatic lesions: for lesions near the body surface, seeds with activity ~1.85 × 10^7^ Bq were used; for bone metastases, ~3.33 × 10^7^ Bq; and for other internal lesions, ~2.22–2.96 × 10^7^ Bq. Under CT guidance, the seeds were implanted percutaneously according to the treatment plan. After implantation, a CT scan of the treated area was performed to verify the dose distribution. If any regions received insufficient dose, additional seeds were implanted based on the verification results.

Anlotinib Treatment: Anlotinib was administered at 12 mg orally, once daily on an empty stomach in the morning, for 14 consecutive days followed by a 7-day rest (21 days per cycle). Treatment was continued in repeating cycles until disease progression (PD) or intolerable toxicity. Dose reductions to 10 mg or 8 mg were permitted based on the patient’s tolerance and observed side effects. If a patient experienced Grade 3 or 4 adverse events (AEs), the dose was reduced according to guidelines (17), and once reduced, the dose was not re-escalated. In the combination treatment group (Group C), anlotinib therapy was initiated on the same day that ^125^I seed implantation was completed.

### 2.4 Data collection and efficacy evaluation

Clinical data collection: Baseline clinical characteristics were recorded, including age, gender, body mass index (BMI), histopathological type, surgical method (extent of thyroidectomy and lymph node dissection), presence of bilateral tumors, presence of extrathyroidal extension, type of tumor metastasis (sites involved), pretreatment performance status (Eastern Cooperative Oncology Group [ECOG] score and Karnofsky Performance Status [KPS]), location of lesions, number of prior ¹³¹I therapies and cumulative ¹³¹I dose, number of target lesions, and the number and total activity of ^125^I seeds implanted.

Follow-up: Patients were regularly followed at 2, 4, and 6 months after treatment, and every 6 months thereafter. Follow-up assessments included documentation of anlotinib usage (ongoing or any interruptions), evaluation of clinical symptoms, routine blood cell counts, liver and kidney function tests, thyroid function tests (FT_3_, FT_4_, TSH, Tg, TgAb), and imaging to assess tumor size. Tumor volumes were calculated from CT images by measuring the three maximum perpendicular diameters of the target lesion: the anteroposterior (a), craniocaudal (b), and transverse (c) diameters. Volume was calculated as *V* = π × *a* × *b* × *c*/6. The volume reduction rate (VRR) was defined as ((original volume – current volume)/original volume) × 100%.

Efficacy evaluation: Tumor size changes were assessed by comparing the maximum lesion diameters (or the short axis for lymph node metastases) and tumor volumes before and after treatment. Serum Tg and TgAb levels were tracked as serological indicators of response to therapy. Pain intensity (for those with bone metastasis-related pain) was measured using the Numerical Rating Scale (NRS) before and after treatment. Treatment-related adverse events (such as pneumothorax, hemorrhage, hypertension, etc.) were recorded in detail and graded according to the National Cancer Institute Common Terminology Criteria for Adverse Events (CTCAE) version 5.0 (18).

Tumor response criteria: Tumor response was determined according to Response Evaluation Criteria in Solid Tumors (RECIST) version 1.1 (19).

• Complete response (CR): Disappearance of all target lesions (and any pathological lymph nodes reduced to < 10 mm short axis), sustained for at least 4 weeks.
• Partial response (PR): At least a 30% decrease in the sum of diameters of target lesions from baseline, sustained for ≥ 4 weeks.
• Progressive disease (PD): At least a 20% increase in the sum of diameters of target lesions (taking the smallest sum on study as reference) or the appearance of new lesions, sustained for ≥ 4 weeks.
• Stable disease (SD): Neither sufficient shrinkage to qualify for PR nor sufficient increase to qualify for PD, with stability maintained for ≥ 4 weeks.

From these, the objective response rate (ORR) was calculated as (CR + PR)/total cases × 100%, and the disease control rate (DCR) as (CR + PR + SD)/total cases × 100%.

Endpoints: The primary endpoint of the study was local progression-free survival (LPFS), defined as the time from the start of treatment to the first occurrence of disease progression (local progression) or death from any cause. Secondary endpoints included overall survival (OS), defined as the time from the start of treatment to death or last follow-up.

### 2.5 Statistical analysis

Statistical analyses were performed using SPSS version 27.0. Continuous data conforming to a normal distribution were expressed as mean ± standard deviation (X^-^ ± *s*) and compared among the three groups using one-way analysis of variance (ANOVA). Continuous data with a non-normal distribution were expressed as median (P_25_, P_75_) and compared using the Kruskal–Wallis test. Categorical data were described as counts and percentages *n* (%). For between-group comparisons of categorical data, if the expected frequency in any cell was < 5 (for unordered categories), Fisher’s exact test was used; for ordered categorical data with frequency < 5, the Kruskal–Wallis test was applied. For within-group comparisons before vs. after treatment: for ≥ 3 time points of non-normally distributed data, the Friedman test was used; for paired two-time-point comparisons, the Wilcoxon signed-rank test was employed. Survival curves were generated using the Kaplan–Meier method, and differences between groups were evaluated by the log-rank test. A two-sided *p* < 0.05 was considered statistically significant.

## 3 Results

### 3.1 Baseline assessment

The median follow-up duration for the 52 patients was 25.5 months (range, 6–88 months), ending on February 28, 2025. Group A consisted of 14 patients who received anlotinib only, Group B included 25 patients who underwent ^125^I seed implantation only, and Group C included 13 patients who received the combination of ^125^I seed implantation and anlotinib. The three groups were similar in their baseline demographic and clinical characteristics, including age, gender, BMI, ECOG score, KPS score, histological subtype (PTC vs. FTC), surgical treatment method, presence of bilateral tumors, presence of extrathyroidal extension, sites of metastasis, and oncogene mutation status; none of these showed a significant difference across groups (all *p* > 0.05) (**Table 1**). In total, 2,795 of the ^125^I seeds were implanted among patients in Groups B and C. The median number of seeds implanted was 56 (interquartile range [IQR]: 40–108) in Group B and 40 (IQR: 15–77.5) in Group C, and this difference was not statistically significant (*p* = 0.26) (**Table 1**).

**Table 1:** Patients’ characteristics.

Characteristics	Group A	Group B	Group C	*P-*value
	N=14	N=25	N=13	
Gender				0.529
Male	7 (50%)	8 (32%)	4 (31%)	
Female	7 (50%)	17 (68%)	9 (69%)	
Age				0.342
Median	52.79 ± 17.27	60.60 ± 15.07	57.85 ± 15.61	
BMI				0.138
Median	21.37 ± 2.34	22.74 ± 3.44	20.71 ± 3.16	
KPS				0.410
Median	90 (90,90)	90 (90,90)	90 (85,90)	
ECOG				0.589
0	10 (71%)	21 (84%)	11 (85%)	
1	4 (29%)	4 (16%)	2 (15%)	
Pathology				0.195
PTC	11 (79%)	12 (48%)	7 (54%)	
FTC	3 (21%)	13 (52%)	6 (46%)	
Surgical methods				0.054
NTT	2 (14%)	1 (4%)	1 (8%)	
TT	1 (7%)	12 (48%)	6 (46%)	
TT + BCLND+LND	11 (77%)	12 (48%)	6 (48%)	
Bilateral tumors				0.152
Yes	7 (50%)	8 (32%)	2 (15%)	
No	7 (50%)	17 (68%)	11 (85%)	
Extrathyroidal extension				0.651
Yes	4 (29%)	8 (32%)	6 (46%)	
No	10 (71%)	17 (68%)	7 (54%)	
Metastasis				0.234
Lymph node only	4 (29%)	4 (16%)	2 (15%)	
Lung only	7 (50%)	6 (24%)	5 (39%)	
Bone only	0 (0%)	8 (32%)	2 (15%)	
Lung and Bone	3 (21%)	7 (28%)	4 (31%)	
Mutations				0.453
Negative	6 (43%)	14 (56%)	3 (23%)	
BRAF V600E alone	5 (36%)	7 (28%)	4 (31%)	
TERT mutation alone	2 (14%)	3 (12%)	4 (31%)	
BRAF+TERT mutations	1 (7%)	1 (4%)	2 (15%)	

BMI stands for Body Mass Index; ECOG stands for Eastern Cooperative Oncology Group Performance Status; KPS stands for Karnofsky Performance Status;PTC stands for Papillary Thyroid Carcinoma;FTC stands for Follicular Thyroid Carcinoma;NTT stands for Near-total Thyroidectomy; TT stands for Total Thyroidectomy; BCLND stands for Bilateral Central Lymph Node Dissection; LND stands for Lateral Lymph Node Dissection.

### 3.2 Efficacy and adverse reactions

Tumor response: At 6 months after treatment, the combination therapy (Group C) produced a significantly higher objective response rate and disease control rate compared to the monotherapy groups. Specifically, 77% of Group C patients achieved an objective response (CR or PR) by 6 months, versus 21% in Group A and 32% in Group B (*p* < 0.05 for both ORR and DCR differences among groups). By 12 months after treatment, however, no statistically significant differences in ORR or DCR were observed among the three groups (**Table 2**).

**Table 2:** Efficacy analysis of treatment at different time points before and after treatment.

Follow-up time	CR	PR	SD	PD	*P-*value	ORR	DCR
6 months after treatment					0.006		
Group A (N=14)	0 (0%)	3 (22%)	10 (71%)	1 (7%)		21% (3/14)	93% (13/14)
Group B (N=25)	0 (0%)	8 (32%)	17 (68%)	0 (0%)		32% (8/25)	100% (25/25)
Group C (N=13)	0 (0%)	10 (77%)	3 (23%)	0 (0%)		77% (10/13)	100% (13/13)
12 months after treatment					0.096		
Group A (N=9)	1 (12%)	2 (22%)	4 (44%)	2 (22%)		33% (3/9)	78% (7/9)
Group B (N=18)	4 (22%)	6 (33%)	7 (39%)	1 (6%)		56% (10/18)	94% (17/18)
Group C (N=13)	4 (30%)	7 (54%)	1 (8%)	1 (8%)		85% (11/13)	92% (12/13)

Data are shown as n (%) ORR = (CR + PR)/total DCR = (CR + PR + SD)/total. CR, complete response; PR, partial response; PD, progressive disease; SD, stable disease; ORR, objective response rate.

Bold values denote p-values derived from the Kruskal-Wallis test for comparing intergroup differences, with p< 0.05 indicating statistical significance.

Tumor size and volume reduction: There were no significant differences in tumor size among the three groups at baseline. All groups showed a reduction in tumor size at 6 months and 12 months post-treatment. At the 6-month evaluation, Group C had a greater median tumor volume reduction than Groups A or B, and the intergroup difference in tumor volume was statistically significant at that time point (*p* = 0.011). Although Group C’s median tumor volume remained lower than those of the other groups at 12 months, the differences at 12 months were not significant (*p* = 0.227). A similar pattern was seen in volume reduction rate (VRR): Group C demonstrated a markedly higher median VRR at 6 months compared to the other groups (*p* = 0.026), but by 12 months the VRR differences were no longer significant (*p* = 0.716). These findings suggest that the combined therapy yielded a more pronounced tumor reduction within the first six months of treatment, and while it continued to be effective at one year, the gap in efficacy between the combination and monotherapies narrowed over time.

Serum thyroglobulin levels: Baseline serum Tg levels were not significantly different across Groups A, B, and C (*p* = 0.104). By 2, 4, and 6 months after treatment, Tg levels had declined significantly from baseline in all three groups. Intergroup comparisons showed that Group C had significantly greater Tg reductions at those time points (2, 4, and 6 months) compared to the other groups (*p* < 0.05 at each of these time points). At 12 months after treatment, serum Tg in Group C had risen slightly (though still below baseline), and there were no significant differences in Tg levels among the groups at the 12-month mark (*p* = 0.542).

Pain scores: Many patients in Group A had no bone metastases and therefore no pain at baseline. In Groups B and C (which included patients with bone metastases), cancer-related pain improved substantially following treatment. NRS pain scores in both Group B and Group C decreased significantly after therapy compared to baseline (within-group p < 0.001 for both), reflecting effective pain palliation. At baseline, Group C had higher pain scores on average than Group B (since more Group C patients had painful bone lesions), and even at 6 months post-treatment, the median pain score in Group C remained slightly higher than in Group B (though both were much improved from baseline), with this difference being statistically significant (*p* < 0.01). Importantly, no new or unexpected pain symptoms were introduced by any of the treatments (apart from transient mild hand–foot skin reactions in some patients on anlotinib).

Adverse events: Treatment-related adverse reactions in all three groups were generally mild and manageable. The observed adverse events included radiation pneumonitis, hypoproteinemia, diarrhea, and myelosuppression, most of which were Grade 1–2 in severity. Two patients in Group A experienced notable events: one had Grade 3 diarrhea and another had severe hypocalcemia; both cases were managed with prompt intervention (including temporary drug discontinuation and appropriate supportive care), and the patients recovered without serious sequelae. There were no treatment-related deaths. The incidence of adverse events did not differ significantly among Groups A, B, and C (*p* = 0.98 for overall comparison; see **Table 3** for details). No patient in Group C had to discontinue the combination therapy due to adverse effects, and any serious AEs that occurred were effectively managed. Overall, adding ^125^I brachytherapy to anlotinib did not appear to exacerbate toxicity compared to anlotinib alone.

**Table 3:** Comparison of changes in related indicators before and after treatment.

	Group A	Group B	Group C	*p-*value
	N=14	N=25	N=13	
Tumor size				
Before treatment	10.54 (11.94,60.56)	78.73 (44.20,141.31)	34.99 (18.13,164.52)	0.540
After 6 months of treatment	5.06 (7.97,22.28)	38.99 (10.78,90.37)	16.21 (4.38,25.69)	**0.011**
After 12 months of treatment	2.24 (2.03,38.86)	23.00 (1.21,89.94)	3.68 (0.00,20.78)	0.227
*p-*value	**0.009**	**<0.001**	**<0.001**	
VRR (%)				
After 6 months of treatment	37.07 (7.45,70.63)	21.52 (14.38,45.79)	78.85 (55.90,91.81)	**0.026**
After 12 months of treatment	42.04 (0.29,89.27)	48.15 (18.75,98.53)	90.57 (78.57,99.94)	0.716
Serum Tg level				
Before treatment	206.20 (23.02,418.20)	305.40 (86.63,4079.00)	827.30 (157.97,5408.25)	0.104
After 2 months of treatment	38.27 (5.46,92.61)	246.70 (25.78,1588.00)	552.20 (51.22,3598.00)	**0.033**
After 4 months of treatment	28.17 (5.88,103.83)	267.60 (21.35,2130.90)	86.18 (31.09,181.25)	**0.040**
After 6 months of treatment	32.59 (2.15,125.49)	260.70 (19.59,2186.88)	56.25 (11.62,128.90)	**0.022**
After 12 months of treatment	25.29 (2.32,132.21)	100.00 (5.77,226.30)	239.00 (24.08,429.40)	0.542
*p-*value	0.108	**<0.001**	**<0.001**	
NRS pain scores				
Before treatment	0.00 (0.00,0.00)	2.00 (0.00,4.00)	4.00 (2.25,5.75)	**<0.001**
After 6 months of treatment	0.00 (0.00,0.00)	1.00 (0.00, 1.00)	2.00 (1.25,2.75)	**<0.001**
	0.317	**<0.001**	**0.004**	
Adverse events (CTCAE 5.0)				0.976
0	9 (64.28%)	13 (52.00%)	7 (53.85%)	
1	2 (14.29%)	10 (40.00%)	5 (38.46%)	
2	1 (7.14%)	2 (8.00%)	1 (7.69%)	
3	2 (14.29%)	0 (0%)	0 (0%)	

VRR, Volume Reduction Rate; NRS, Numerical Rating Scale.

### 3.3 LPFS and OS

**Figure 2:**
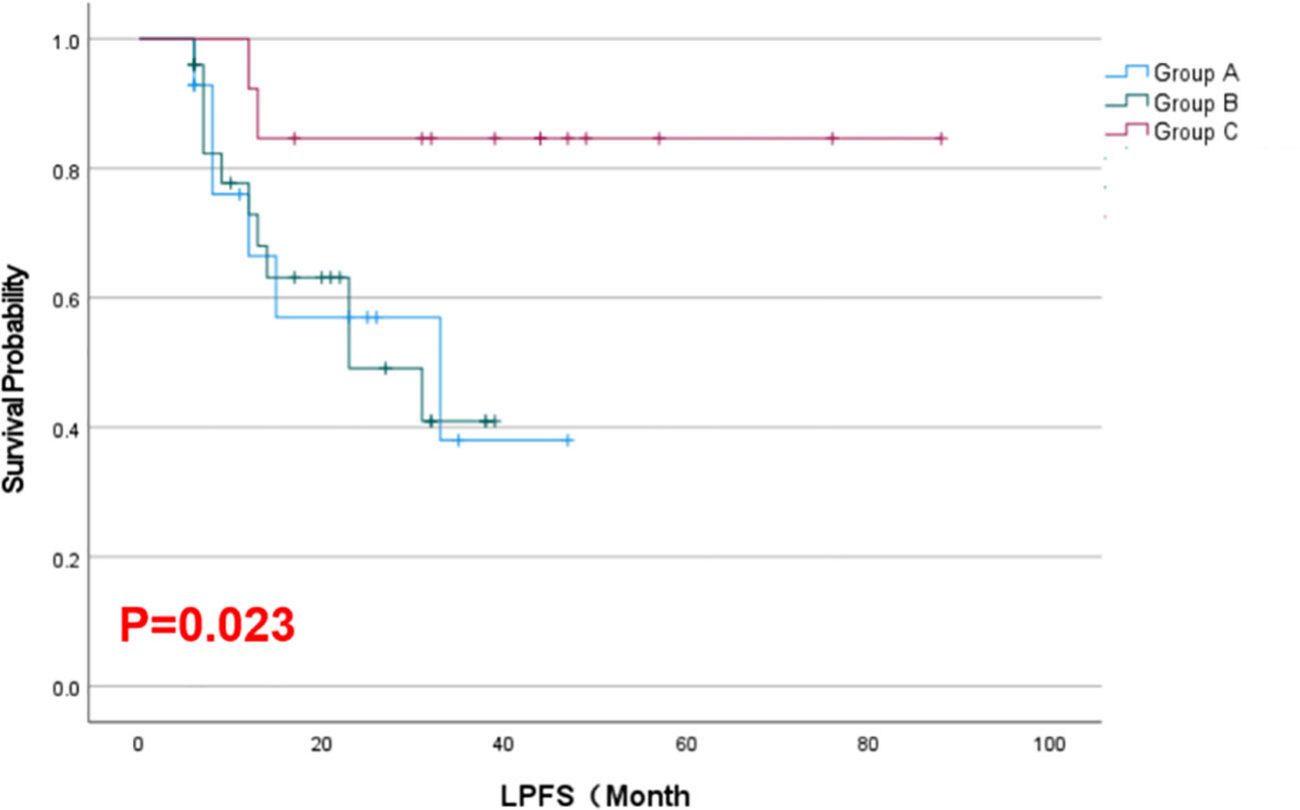
Kaplan–Meier curves for LPFS in the three groups of patients.

**Figure 3:**
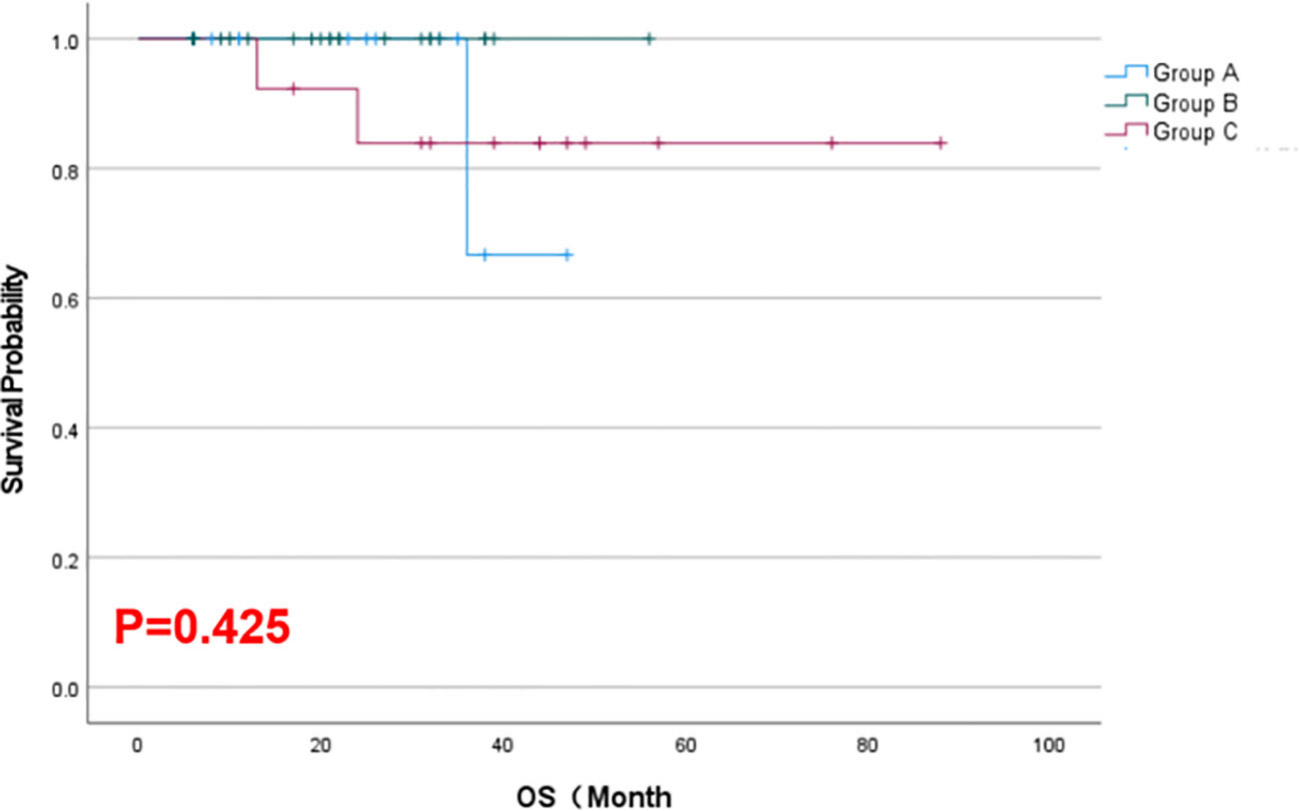
Kaplan–Meier curves for OS in the three groups of patients.

As of February 2025, the mean local progression-free survival was 18.64 months in Group A, 18.68 months in Group B, and 42.23 months in Group C. The Kaplan–Meier survival curves for LPFS demonstrated a significant prolongation of LPFS in the combination group (Group C) compared to the monotherapy groups (*p* = 0.023, **Figure 2**). The mean overall survival was 22.21 months in Group A, 22.40 months in Group B, and 43.15 months in Group C. Although Group C showed a numerically longer OS than Groups A and B, this difference was not statistically significant by Kaplan–Meier analysis (*p* = 0.425, **Figure 3**). These results indicate that, compared with single-agent treatment, the combination of ^125^I brachytherapy and anlotinib significantly extended local PFS but did not significantly improve OS within the observed follow-up period.

During follow-up, we also examined causes of death and patterns of disease progression in each group. In Group A, a total of 3 patients died: 2 deaths were due to severe influenza A infection and 1 was due to distant disease progression. In Group B, 1 patient died from a severe pulmonary infection. In Group C, 2 patients died: one due to cardiac tamponade (attributed to tumor involvement) and the other due to local tumor progression. Notably, none of the deaths in any group were caused by treatment-related adverse reactions.

## 4 Discussion

Advanced RAIR-DTC poses a major therapeutic challenge, especially for patients with symptomatic, rapidly progressing, locally advanced or widely metastatic disease that is not amenable to surgery. Systemic targeted therapies can significantly improve clinical outcomes in RAIR-DTC (20), and TKIs in particular have become the standard of care for progressive disease (1, 21). Meanwhile, ^125^I seed brachytherapy has emerged as a preferred modality for local tumor control due to its excellent local efficacy, minimally invasive nature, and favorable safety profile (22). In this study, we explored a combined modality treatment aimed at maximizing tumor control by addressing both systemic disease (with anlotinib) and local tumor burden (with ^125^I brachytherapy).

**Figure 4:**
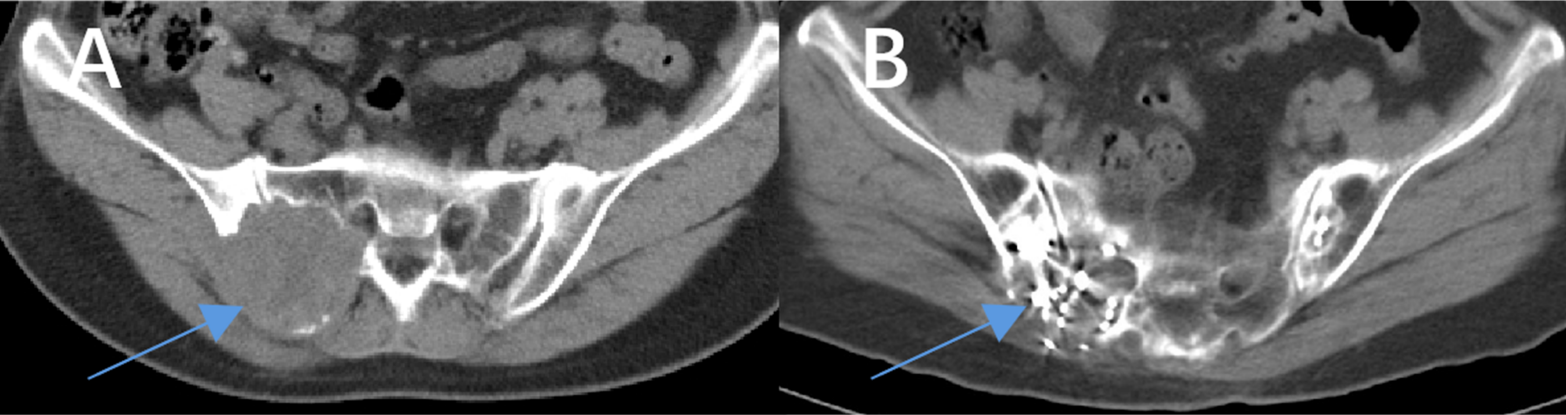
Comparison of images before and after treatment with anlotinib combined with ^125^I seed implantation in a patient with iodine-refractory follicular thyroid carcinoma. The patient had been taking anlotinib as prescribed since July 2020. A total of 77 seeds were implanted in two sessions during this period. **(A)** shows the maximum cross-sectional CT image of the lesion before treatment (the arrow indicates the lesion), and **(B)** shows the corresponding CT image after treatment. A significant reduction in the lesion size can be seen.

**Figure 5:**
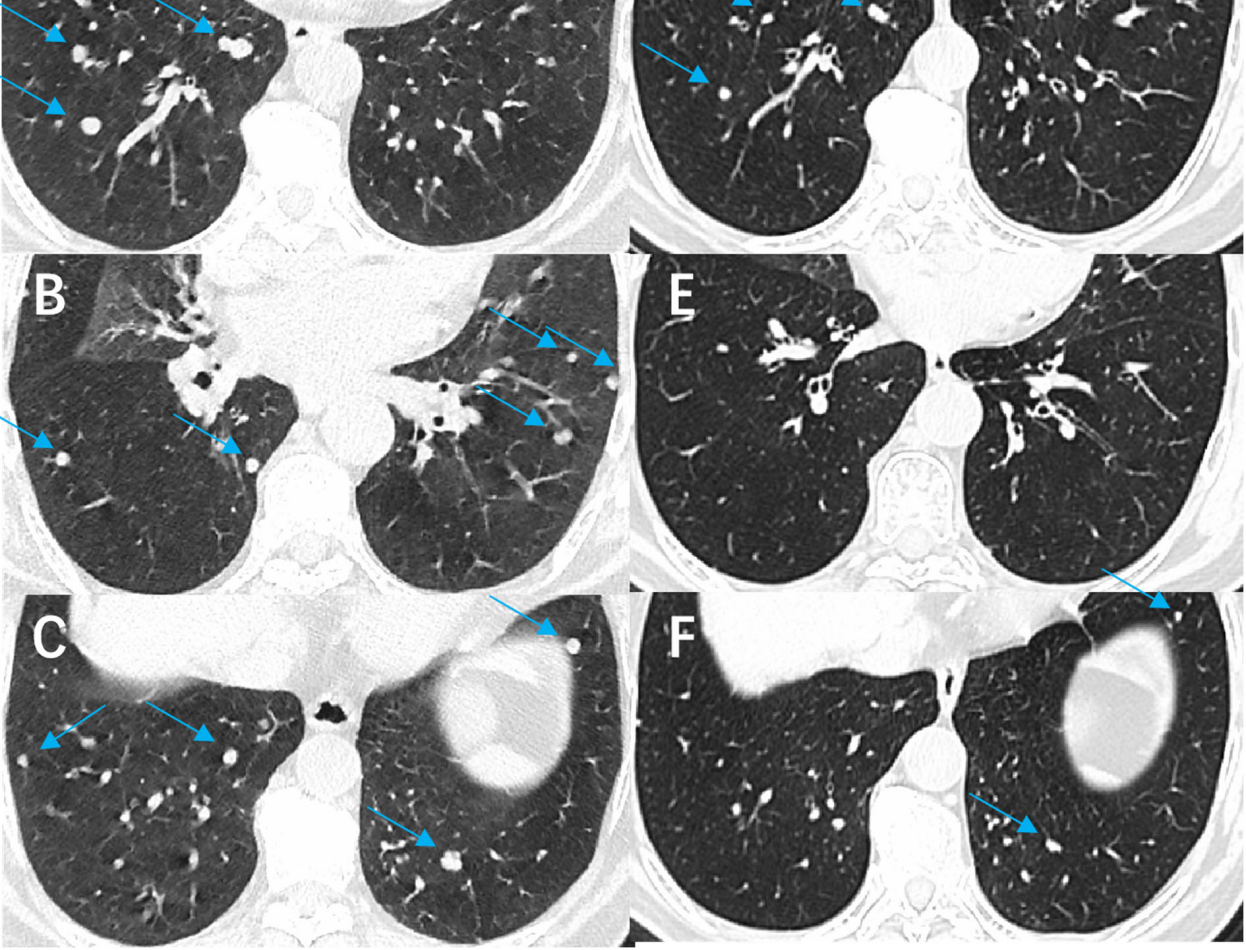
Comparison of images before and after treatment with anlotinib combined with ^125^I seed implantation in a patient with refractory follicular thyroid carcinoma. The patient had been taking anlotinib since March 2022. **(A-C)** show the CT images of both lungs before treatment (arrows indicate the lesions), and **(D-F)** show the corresponding CT images after treatment. A significant reduction in the number and size of pulmonary metastatic lesions is evident.

Our results demonstrate that combining ^125^I seed implantation with anlotinib can achieve superior short-term tumor control compared to either treatment alone. The combination therapy group (Group C) had significantly higher tumor response rates at 6 months and markedly prolonged local PFS relative to the other groups. At the 6-month evaluation, 77% of Group C patients showed objective tumor regression (CR or PR) (**Figure 4**), a response rate much higher than that achieved by anlotinib alone (21%) or brachytherapy alone (32%). Furthermore, we observed instances in the combination group of untreated metastatic lesions (for example, lung nodules that were not implanted with seeds) shrinking or even disappearing during therapy (**Figure 5**). Prior studies have reported the effectiveness of each component individually — for example, Chen et al. (23) achieved symptom relief in 6 of 9 patients by using ^125^I seed implantation for bone metastases from thyroid cancer, and Huang et al. (24) observed an ORR of 76.9% with anlotinib monotherapy in advanced thyroid cancer. Our study builds on these findings by showing that the combination of anlotinib and ^125^I brachytherapy can further improve outcomes beyond what either modality achieves alone.

These findings suggest a synergistic effect, whereby the two modalities together produce greater tumor shrinkage than either could alone. The possible mechanisms of synergy are as follows: ^125^I seeds emit continuous low-energy X-rays and γ-rays that induce DNA damage in tumor cells, leading to G_2_/M cell-cycle arrest, mitotic inhibition, and apoptosis. This greatly reduces tumor cell proliferation, invasiveness, and metastatic potential (22), thereby enhancing the efficacy of anlotinib in controlling the disease (25). Meanwhile, brachytherapy can increase the sensitivity of residual tumor cells to systemic therapies (26). It also inhibits tumor angiogenesis by downregulating HIF-1α and VEGF expression (27), exerting an additive anti-angiogenic effect that complements anlotinib’s inhibition of VEGF/VEGFR pathways.

The improved local control with combination therapy was clearly reflected in the LPFS outcomes. Group C’s median LPFS (~42 months) was more than double that of the monotherapy groups (~18.6 months). This indicates that adding brachytherapy to systemic treatment can substantially delay local progression of disease. From a clinical perspective, prolonging LPFS is meaningful as it can translate into longer periods of symptom relief and a reduced need for palliative interventions at sites of bulky disease.

However, it is noteworthy that the combination did not significantly prolong OS in our cohort. This is likely due to the relatively short follow-up duration and the fact that once the disease progresses systemically, patients may receive other salvage treatments which can even out survival across groups. It is also possible that a true OS benefit from the combination might emerge with longer observation; indeed, we observed a numerical advantage in median OS for Group C (43.1 months) versus the others (~22.3 months), although this difference did not reach statistical significance in our sample. Larger studies with extended follow-up will be needed to determine whether the combination confers a real OS benefit.

Despite the clear early benefits, our data also show that by one year post-treatment, the differences between the combination and monotherapy groups had diminished in terms of ORR, tumor volume reduction, and Tg levels. Additionally, as mentioned, there was no significant improvement in long-term OS with the combined approach. These observations underscore that RAIR-DTC remains fundamentally a systemic disease problem—while local control was improved, the overall disease course (including the eventual development of new distant metastases or drug resistance) still limited patient survival.

Therefore, the combined modality should be viewed as a means to achieve better disease control and extend the duration of local remission, rather than a definitive cure. It provides patients a longer period with their disease under control locally, which is valuable for symptom management and quality of life, but ultimately the impact on survival may be limited unless systemic disease control can be further improved with additional or subsequent therapies.

An important goal in treating advanced thyroid cancer is to improve patient symptoms and quality of life. Bone metastases from thyroid cancer often cause severe pain and skeletal complications. The continuous low-dose radiation from ^125^I seeds not only kills tumor cells and reduces their compression of surrounding nerves or organs, but also inhibits the release of pain-inducing factors by tumor cells (28). Consistent with previous studies (29–31), we found that ^125^I seed brachytherapy was highly effective for pain palliation. Most patients with bone pain in our study reported immediate or early relief after seed implantation. Both Group B and Group C showed significant reductions in NRS pain scores following treatment, reflecting improved patient comfort and daily functionality. In some cases, the pain relief was dramatic—patients who were opioid-dependent at baseline became free of pain medication after brachytherapy, and many were pain-free on subsequent follow-ups.

It is worth mentioning that effective pain control can indirectly allow patients to remain more active and maintain a better performance status, thereby enabling them to tolerate systemic therapy for longer durations. In this sense, the combination of anlotinib with local therapy contributed to quality-of-life improvements by both reducing tumor burden and alleviating cancer-related symptoms. (Patients in Group A mostly had no pain at baseline due to differences in metastatic sites, so direct comparison of pain outcomes between Group A and the other groups is limited.) Importantly, none of the treatment regimens in our study introduced new chronic pain issues (aside from transient hand–foot skin reaction discomfort in some patients on anlotinib), indicating that the therapies did not adversely affect pain status.

The safety profile observed with the combined anlotinib and ^125^I seed therapy was favorable. The types of adverse reactions were similar to those seen with each treatment independently, and most were low grade. The majority of patients in all groups experienced only Grade 1–2 side effects, which were manageable with standard supportive care. This suggests that ^125^I seed implantation can be added to systemic therapy without introducing significant overlapping toxicities, likely because one modality acts locally and the other systemically.

Comparing our safety findings to the literature, previous studies of anlotinib monotherapy in thyroid cancer reported substantial rates of hypertension, hand–foot syndrome, and other TKI-associated AEs (21). In our cohort, the incidence of these AEs in the combination group (Group C) was not higher than in the anlotinib-only group (Group A). No patients discontinued the combined therapy due to adverse effects, all serious AEs that did occur were effectively managed with appropriate interventions, and patients recovered without permanent harm. We observed no treatment-related deaths and no late complications attributable to brachytherapy during the follow-up period. These results reinforce that the combination approach is well tolerated. This is crucial because RAIR-DTC patients have limited treatment options; a combined therapy would only be viable in practice if it does not compromise safety or quality of life. Our study provides evidence that the anlotinib + ^125^I seed regimen meets this requirement, with toxicity that is mild and comparable to monotherapy.

It is also worth noting that the absence of severe high-grade toxicities in our series may be partly due to careful patient monitoring and proactive management. For example, regular laboratory tests allowed early detection of issues such as liver enzyme elevations or cytopenias, and prompt interventions prevented these from progressing to higher-grade events. Blood pressure was closely monitored and controlled to mitigate Grade 3 hypertension. By adhering to dose modification guidelines, we minimized the risk of serious adverse outcomes. These measures underscore that strict monitoring and comprehensive supportive care are integral to safely combining these treatments.

**Figure 6:**
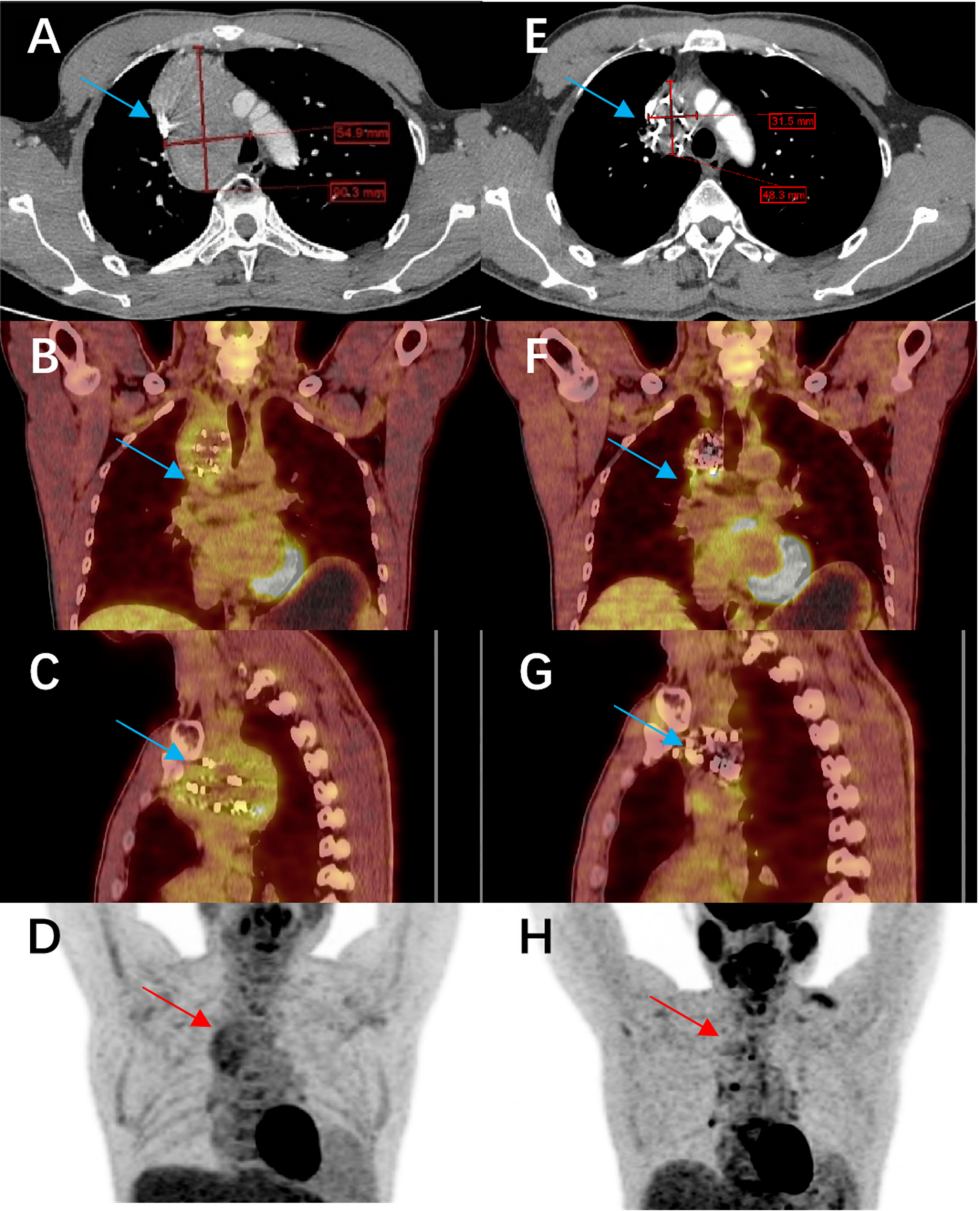
Comparison of imaging before and after treatment with anlotinib combined with ^125^I seed implantation in a patient with refractory papillary thyroid carcinoma. The patient had been on anlotinib since March 2021, and a total of 140 seeds were implanted. **(A–D)** show the pre-treatment CT and PET-CT images of the lesion (cross-sectional views), and **(E–H)** show the corresponding post-treatment CT and PET-CT images. The lesion has significantly shrunk, and its metabolic activity has markedly decreased.

One interesting observation from our study relates to metabolic tumor activity. In some Group C patients who underwent ¹^8^F-FDG PET/CT imaging, we documented a significant decrease in metabolic activity (FDG uptake) in tumor lesions after the combined therapy. This suggests that beyond anatomical tumor shrinkage, the combination therapy may also induce functional changes in tumors, potentially reflecting reduced tumor viability or altered tumor biology. We did not systematically include PET response as an endpoint (because not all patients had PET/CT scans), but these anecdotal findings hint at an additional dimension of treatment effect. **Figure 6** illustrates one such case: the PET/CT images before and after treatment show marked reductions in both tumor size and metabolic activity in a refractory thyroid carcinoma patient treated with anlotinib plus ^125^I seeds. This raises the possibility that metabolic response might precede anatomic response and could serve as an early indicator of treatment effectiveness. Future studies might incorporate PET-based metabolic response criteria as an additional outcome measure to further quantify tumor response to the combined modality therapy.

This study has several limitations. First, it was a retrospective analysis with a relatively small sample size, especially for the combination therapy group (only 13 patients). The non-randomized design and modest cohort size may introduce selection bias and limit the statistical power to detect certain differences between groups. Second, the follow-up duration (median ~25.5 months) may be insufficient to fully assess long-term outcomes such as 5-year survival or late safety events. RAIR-DTC can be indolent in some patients, and longer follow-up will be needed to determine if the early benefits of combination therapy translate into extended survival or durable disease control. Third, due to the retrospective design, some data—such as detailed patient-reported quality of life measures—were not collected systematically; our analysis of symptom outcomes like pain relied on clinical records rather than standardized questionnaires.

There are also technical considerations with ^125^I seed therapy that impose limitations. Brachytherapy is most suitable for tumors in locations accessible to percutaneous needle implantation; lesions in certain anatomical sites (for example, deep mediastinal lymph nodes adjacent to the heart or great vessels) may not be feasible to treat with seeds. In our series, patients with disease in such difficult-to-access locations were underrepresented, so our results apply primarily to those with implantable lesions. Additionally, factors such as non-uniform seed distribution, radiation attenuation by dense tissues (e.g., bones), and patient-specific anatomy can affect the efficacy of brachytherapy. Despite careful pre-planning, some patients might receive suboptimal dosing if seeds cannot be ideally placed—for instance, tumors that move with respiration in the lungs or mediastinum can pose a challenge for accurate implantation. These factors may lead to variability in brachytherapy outcomes and should be considered when selecting patients for this approach.

## 5 Conclusion

For patients with advanced RAIR-DTC, ^125^I seed implantation combined with anlotinib targeted therapy offers a promising treatment option. Our study suggests that this combined modality can effectively control both local and systemic aspects of the disease: it achieved greater tumor shrinkage, higher response rates, and significantly prolonged local progression-free survival compared to either modality alone, while maintaining a favorable safety profile. Patients receiving the combination therapy experienced relief of tumor-related symptoms (such as bone pain) and improvements in quality of life, with adverse reactions that were few, mostly mild, and manageable. Therefore, anlotinib plus ^125^I brachytherapy may be considered as a therapeutic strategy for RAIR-DTC, especially in cases with bulky or inoperable lesions that are resistant to radioiodine.

## Data Availability

The original contributions presented in the study are included in the article/**Supplementary Material**. Further inquiries can be directed to the corresponding author.

## References

[1] Vaccher E Schioppa O Martellotta F Fornasier G Giacomin E Re L. Safety profiles and pharmacovigilance considerations for recently patented anticancer drugs: advanced thyroid cancer. Recent Pat Anticancer Drug Discov. (2019) 14:226–41. doi: 10.2174/1574892814666190726143011, PMID: 31362663

[2] Zheng RS Chen R Han BF Wang SM Li L Sun KX. Cancer incidence and mortality in China, 2022. Zhonghua Zhong Liu Za Zhi. (2024) 46:221–31. doi: 10.3760/cma.j.cn112152-20240119-00035, PMID: 38468501

[3] Laha D Nilubol N Boufraqech M. New therapies for advanced thyroid cancer. Front Endocrinol (Lausanne). (2020) 11:82. doi: 10.3389/fendo.2020.00082, PMID: 32528402 PMC7257776

[4] Do Cao C Godbert Y Bardet S Borson-Chazot F Decaussin-Petrucci M Wassermann J. ENDOCAN-TUTHYREF network. ENDOCAN TUTHYREF network consensus recommendations: Refractory follicular-derived thyroid cancer. Ann Endocrinol (Paris). (2025) 86:101735. doi: 10.1016/j.ando.2025.101735, PMID: 40345488

[5] Sprindzuk MV. Angiogenesis in Malignant thyroid tumors. World J Oncol. (2010) 1:221–31. doi: 10.4021/wjon263e, PMID: 29147212 PMC5649747

[6] Albarrán V Villamayor ML Chamorro J Rosero DI Pozas J San Román M. Receptor tyrosine kinase inhibitors for the treatment of recurrent and unresectable bone sarcomas. Int J Mol Sci. (2022) 23:13784. doi: 10.3390/ijms232213784, PMID: 36430263 PMC9697271

[7] Li J Han N Lu CH Wang CC Zhao ZL Wang H. Analysis of the efficacy and safety of anlotinib in the treatment of distant metastatic radioactive iodine-refractory differentiated thyroid cancer. Chin J Nucl Med Mol Imaging. (2023) 43:470–4. doi: :10.3760/cma.j.issn.2095-2848.2018.01.004

[8] Bai J Yu Q Wang Y Xu L Wang J Zhai J. Iodine-125 brachytherapy suppresses tumor growth and alters bone metabolism in a H1299 xenograft mouse model. Med Oncol. (2023) 40:72. doi: 10.1007/s12032-022-01937-z, PMID: 36607460

[9] Fan X Liang X Guo Y Jin G Ming D An X. ^125^I inhibits the progression of cervical cancer by upregulating the HSF1/PU.1/SYK signaling pathway and consequently enhancing the apoptotic response mediated by ROS/USP7/P53. Sci Rep. (2025) 15:17690. doi: 10.1038/s41598-025-99214-2, PMID: 40399470 PMC12095467

[10] Zhang W Hao S Wang Z Ding T Zhang G. ^125^I seed implantation for lymph node metastasis from radioactive iodine-refractory differentiated thyroid carcinoma: a study on short-term efficacy and dosimetry. Front Oncol. (2024) 14:1325987. doi: 10.3389/fonc.2024.1325987, PMID: 38988713 PMC11233436

[11] Wan Q Tan L Tang X Wang W Su Y Wu Z. The clinical value of iodine-125 seed implantation in the treatment of iodine-refractory differentiated thyroid carcinoma. Front Endocrinol (Lausanne). (2024) 15:1327766. doi: 10.3389/fendo.2024.1327766, PMID: 38686207 PMC11056783

[12] Su Y Wang J Huang L Xie L Yu X Zha J. Clinical efficacy of iodine-125 (^125^I) seed implantation in patients with iodine-refractory differentiated thyroid cancer. Am J Cancer Res. (2023) 13:4794–802., PMID: 37970343 PMC10636672

[13] Zhang W Guo L Hao S Wang Z Ji L Ge X. Clinical value of ^125^I radioactive seed implantation in the treatment of lymph node metastasis of ¹³¹I-refractory differentiated thyroid cancer. Appl Radiat Isot. (2023) 199:110868. doi: 10.1016/j.apradiso.2023.110868, PMID: 37392614

[14] Shi J Zhang Y Wang J Li J Li Z. Anlotinib combined with chemoradiotherapy exhibits significant therapeutic efficacy in esophageal squamous cell carcinoma. Front Oncol. (2020) 10:995. doi: 10.3389/fonc.2020.00995, PMID: 32754439 PMC7365939

[15] Tang G Zhang Y Meng W Zhong S Feng H Yu G. Iodine-125 seeds combined with anlotinib in the treatment of recurrent retroperitoneal liposarcoma after surgery: a case report. Front Oncol. (2025) 15:1540868. doi: 10.3389/fonc.2025.1540868, PMID: 40356749 PMC12066600

[16] Nuclear Medicine Expert Committee of Chinese Society of Clinical Oncology, Thyroid Cancer Expert Committee of Chinese Society of Clinical Oncology, Chinese Society of Nuclear Medicine of Chinese Medical Association, Thyroid Disease Professional Committee of Chinese Research Hospital Association, Thyroid Disease Branch of China International Exchange and Promotion Association for Medical and Health Care, Thyroid Cancer Professional Committee of Chinese Anti-Cancer Association. Guidelines for the diagnosis, treatment and management of radioactive iodine-refractory differentiated thyroid cancer (2024 edition). Chin J Nucl Med Mol Imaging. (2024) 44:359–72. doi: 10.3760/cma.j.cn321828-20240125-00034

[17] Chi Y Zheng X Zhang Y Shi F Cheng Y Guo Z. Anlotinib in locally advanced or metastatic radioiodine-refractory differentiated thyroid carcinoma: A randomized, double-blind, multicenter phase II trial. Clin Cancer Res. (2023) 29:4047–56. doi: 10.1158/1078-0432.CCR-22-3406, PMID: 37594724 PMC10570678

[18] Freites-Martinez A Santana N Arias-Santiago S Viera A. Using the common terminology criteria for adverse events (CTCAE) version 5.0 to evaluate the severity of adverse events of anticancer therapies. Actas Dermosifiliogr (Engl Ed). (2021) 112:90–2. doi: 10.1016/j.ad.2019.05.009, PMID: 32891586

[19] Eisenhauer EA Therasse P Bogaerts J Schwartz LH Sargent D Ford R. New response evaluation criteria in solid tumours: revised RECIST guideline (version 1.1). Eur J Cancer. (2009) 45:228–47. doi: 10.1016/j.ejca.2008.10.026, PMID: 19097774

[20] Zhang Y Zhang X Lin L Xing M. Efficacy and safety of targeted therapy for radioiodine-refractory differentiated thyroid cancer. J Clin Endocrinol Metab. (2025) 110:873–86. doi: 10.1210/clinem/dgae617, PMID: 39292866

[21] Lin YS Wang RF Huang R Wen Q Cao W Chen LB. Chinese management guidelines for radioactive iodine-refractory differentiated thyroid cancer (2025 edition). Eur J Nucl Med Mol Imaging. (2025). doi: 10.1007/s00259-025-07222-1, PMID: 40128355 PMC12316754

[22] Hu P Huang J Zhang Y Guo H Chen G Zhang F. Iodine-125 seed implantation in the treatment of Malignant tumors. J Interv Med. (2023) 6:111–5. doi: 10.1016/j.jimed.2023.07.006, PMID: 37846333 PMC10577067

[23] Chen ZJ Tan LL Su Y Xie JG Zhou AQ Zhou WW. Clinical application of ^125^I seeds implantation for bone metastasis from iodine-refractory differentiated thyroid cancer. Chin J Nucl Med Mol Imaging. (2018) 38:14–6. doi: 10.3760/cma.j.cn321828-20230327-00083

[24] Huang NS Wei WJ Xiang J Chen JY Guan Q Lu ZW. The efficacy and safety of anlotinib in neoadjuvant treatment of locally advanced thyroid cancer: A single-arm phase II clinical trial. Thyroid. (2021) 31:1808–13. doi: 10.1089/thy.2021.0307, PMID: 34610756

[25] Li H Duan Z Zhao C Fang W Jia Y Li X. Combination of brachytherapy with iodine-125 seeds and systemic chemotherapy versus systemic chemotherapy alone for synchronous extracranial oligometastatic non-small cell lung cancer. Cancer Manag Res. (2020) 12:8209–20. doi: 10.2147/CMAR.S267694, PMID: 32982417 PMC7494957

[26] Fabian A Pyschny F Krug D. Lokal konsolidierende Strahlentherapie beim oligometastasierten Nicht-kleinzelligen Bronchialkarzinom [Local consolidative therapy vs. maintenance therapy or observation for patients with oligometastatic non-small-cell lung cancer: long-term results of a multi-institutional, phase II, randomized study. Strahlenther Onkol. (2019) 195:1113–5. doi: 10.1007/s00066-019-01528-4, PMID: 31637448

[27] Xiang GL Zhu XH Lin CZ Wang LJ Sun Y Cao YW. ^125^I seed irradiation induces apoptosis and inhibits angiogenesis by decreasing HIF-1α and VEGF expression in lung carcinoma xenografts. Oncol Rep. (2017) 37:3075–83. doi: 10.3892/or.2017.5521, PMID: 28339070

[28] Jiang AG Lu HY Ding ZQ. Implantation of ^125^I radioactive seeds via c-TBNA combined with chemotherapy in an advanced non-small-cell lung carcinoma patient. BMC Pulm Med. (2019) 19:205. doi: 10.1186/s12890-019-0974-8, PMID: 31703663 PMC6842247

[29] Feng S Wang L Xiao Z Maharjan R Chuanxing L Fujun Z. ^125^I seed implant brachytherapy for painful bone metastases after failure of external beam radiation therapy. Med (Baltimore). (2015) 94:e1253. doi: 10.1097/MD.0000000000001253, PMID: 26252288 PMC4616588

[30] Xiang Z Wang L Yan H Zhong Z Liu W Mo Z. ^125^I seed brachytherapy versus external beam radiation therapy for the palliation of painful bone metastases of lung cancer after one cycle of chemotherapy progression. Onco Targets Ther. (2018) 11:5183–93. doi: 10.2147/OTT.S154973, PMID: 30214224 PMC6118334

[31] Liu Y Zhang C Xu K Wu K Han X Jiao D. ^125^I brachytherapy: a useful treatment to control painful osteoblastic bone metastases. Clin Transl Oncol. (2023) 25:1297–306. doi: 10.1007/s12094-022-03025-0, PMID: 36472748 PMC10119221

