# Supplementary material for: Efficacy and safety of anlotinib combined with 125I seed implantation for iodine-refractory thyroid cancer
Source: Front Endocrinol (Lausanne). 2025 Aug 22;16:1587412. doi: 10.3389/fendo.2025.1587412 (PMC12411173; doi:10.3389/fendo.2025.1587412)
Supplement: Supplementary file 1 [file DataSheet1.docx]

**6.21 Supplementary Material 1**

**Statistical Methods Table of the Article.**

| **Data Type** | **Data Characteristics** | **Statistical Method** | **Corresponding Indicators** |
| --- | --- | --- | --- |
| **Inter-group Comparison** | | | |
| Continuous | Normally Distributed | One-way ANOVA | AGE, BMI |
| Continuous | Non-normally Distributed | Kruskal-Wallis Test | Tumor Size, Serum Tg Level, NRS Pain Scores, VRR |
| Categorical (Unordered) | Cell frequency < 5 | Chi-square Test (Fisher’s Exact Test) | Gender, Bilateral tumors, Surgical methods, Pathology, Extrathyroidal extension, Metastasis, Mutations |
| Categorical (Ordered) | Cell frequency < 5 | Kruskal-Wallis Test | ECOG, Objective clinical remission, Adverse Events |
| **Intra-group Comparison (Before vs. After Treatment)** | | | |
| Continuous (Non-normal) | ≥ 3 groups | Friedman Test | Tumor Size, Serum Tg Level |
| Continuous (Non-normal) | 2 groups | Wilcoxon Paired Test | NRS Pain Score |
| **Survival Analysis** | | | |
| Survival Rate Comparison | Kaplan-Meier Analysis | LPFS/OS |  |
| **Analysis of Independent Predictors** | | | |
|  | Univariate screening | Log-Rank Test |  |
|  | Multivariate analysis | Cox Regression Analysis |  |
